# Supplementary material for: Primary series COVID-19 vaccine effectiveness among health care workers in the country of Georgia, March–December 2021
Source: PLoS One. 2024 Sep 6;19(9):e0307805. doi: 10.1371/journal.pone.0307805 (PMC11379210; doi:10.1371/journal.pone.0307805)
Supplement: S1 Fig — (DOCX) [file pone.0307805.s001.docx]

Figure S1. Whole genome sequencing results of samples from SARS-CoV-2 positive cases in Georgia by week during the study analysis period, 2021*

| **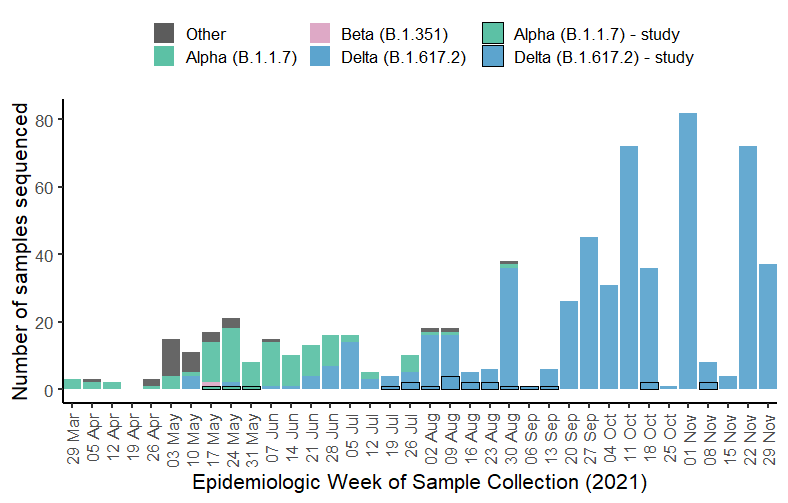** |
| --- |
|  |
|  |
| * Sequencing data from GISAID (N=678) were downloaded on 27 January 2023 and reflect samples collected through general surveillance in Georgia that were collected between 23 March and 5 December, 2021; “Study” samples reflect Whole genome sequencing results from SARS-CoV-2 positive study participants (N=22) from March 29-November 29, 2021 |
