# Supplementary material for: Primary series COVID-19 vaccine effectiveness among health care workers in the country of Georgia, March–December 2021
Source: PLoS One. 2024 Sep 6;19(9):e0307805. doi: 10.1371/journal.pone.0307805 (PMC11379210; doi:10.1371/journal.pone.0307805)
Supplement: S1 File — (DOCX) [file pone.0307805.s002.docx]

**Supporting Tables S1-S5**

| Table S1. Demographic, Occupational and Health Characteristics, Prior Infection Status of Participants in COVID-19 Vaccine Effectiveness study, by vaccination status at the end of the study period, Georgia, 2021 | | | |
| --- | --- | --- | --- |
| **Characteristic/Category** | **All Participants, n=1561** | **Unvaccinated, n=479** | **Vaccinated with primary series (2 doses), n=1082** |
| **Age, n=1561** | | | |
| Median (IQR) | 40 (30-53) | 38 (28-52) | 41 (30-53) |
| **Age group, n=1561** | | | |
| <20, n (%) | 16 (1) | 3 (<1) | 13 (1) |
| 20-29, n (%) | 373 (24) | 138 (29) | 235 (22) |
| 30-39, n (%) | 343 (22) | 105 (22) | 238 (22) |
| 40-49, n (%) | 330 (21) | 94 (20) | 236 (22) |
| 50-59, n (%) | 314 (20) | 90 (19) | 224 (21) |
| 60+, n (%) | 185 (12) | 49 (10) | 136 (13) |
| **Sex, n=1561** | | | |
| female, n (%) | 1318 (84) | 397 (83) | 921 (85) |
| male, n (%) | 243 (16) | 82 (17) | 161 (15) |
| **Hospital, n=1561** | | | |
| Acad. K Central University Hosp., n (%) | 300 (19) | 79 (16) | 221 (20) |
| Batumi Republican Hospital, n (%) | 276 (18) | 77 (16) | 199 (18) |
| Bochorishvili Clinic, n (%) | 194 (12) | 73 (15) | 121 (11) |
| Bokeria Tbilisi Referral Hospital, n (%) | 309 (20) | 101 (21) | 208 (19) |
| Caucasus Medical Centre, n (%) | 299 (19) | 93 (19) | 206 (19) |
| Infectious Disease Hospital, n (%) | 183 (12) | 56 (12) | 127 (12) |
| **Occupation/Role in hospital, n=1561** | | | |
| Nurse or Midwife, n (%) | 604 (39) | 181 (38) | 423 (39) |
| Medical Doctor, n (%) | 306 (20) | 50 (10) | 256 (24) |
| Other, n (%) | 651 (42) | 248 (52) | 403 (37) |
| **Household size, n=1561** | | | |
| 1-3, n (%) | 703 (45) | 220 (46) | 483 (45) |
| 4-5, n (%) | 622 (40) | 187 (39) | 435 (40) |
| 6+, n (%) | 236 (15) | 72 (15) | 164 (15) |
| **Any chronic condition, n=1561** | | | |
| No, n (%) | 1171 (75) | 355 (74) | 816 (75) |
| Yes, n (%) | 390 (25) | 124 (26) | 266 (25) |
| **Number of chronic conditions, n=1561** | | | |
| 0, n (%) | 1171 (75) | 355 (74) | 816 (75) |
| 1, n (%) | 307 (20) | 103 (22) | 204 (19) |
| ≥2, n (%) | 83 (5) | 21 (4) | 62 (6) |
| **Body mass index, n=1561** | | | |
| Underweight or normal, n (%) | 721 (46) | 224 (47) | 497 (46) |
| Overweight, n (%) | 481 (31) | 145 (30) | 336 (31) |
| Obese, n (%) | 359 (23) | 110 (23) | 249 (23) |
| **Smoking, n=1560** | | | |
| Currently/previously smokes, n (%) | 530 (34) | 155 (32) | 375 (35) |
| Never smokes, n (%) | 1030 (66) | 323 (68) | 707 (65) |
| **Self-assessed health status, n=1561** | | | |
| Excellent, n (%) | 127 (8) | 35 (7) | 92 (9) |
| Very good, n (%) | 252 (16) | 77 (16) | 175 (16) |
| Good, n (%) | 521 (33) | 166 (35) | 355 (33) |
| Fair, n (%) | 641 (41) | 191 (40) | 450 (42) |
| Poor, n (%) | 20 (1) | 10 (2) | 10 (<1) |
| **Hands on care, n=1561** | | | |
| No, n (%) | 745 (48) | 268 (56) | 477 (44) |
| Yes, n (%) | 816 (52) | 211 (44) | 605 (56) |
| **Flu vaccine September 2020, n=1561** | | | |
| No, n (%) | 1068 (68) | 389 (81) | 679 (63) |
| Yes, n (%) | 492 (32) | 89 (19) | 403 (37) |
| **Face-to-face patient contact, n=1561** | | | |
| No, n (%) | 1225 (78) | 355 (74) | 870 (80) |
| Yes, n (%) | 336 (22) | 124 (26) | 212 (20) |
| **SARS CoV-2 Vaccine product received prior to the end of the study period, n= 1561** | | | |
| Unvaccinated, n (%) | 344 (22) | 344 (72) | 0 (0) |
| ChAdOx1-S - 1 dose, n (%) | 2 (<1) | 2 (<1) | 0 (0) |
| ChAdOx1-S - 2 doses, n (%) | 26 (2) | 0 (0) | 26 (2) |
| BNT162b2 - 1 dose, n (%) | 87 (6) | 86 (18) | 1 (<1) |
| BNT162b2 - 2 dose, n (%) | 745 (48) | 0 (0) | 745 (69) |
| BBIBP-CorV - 1 dose, n (%) | 34 (2) | 33 (7) | 1 (<1) |
| BBIBP-CorV - 2 dose, n (%) | 238 (15) | 0 (0) | 238 (22) |
| CoronaVac - 1 dose, n (%) | 14 (<1) | 14 (3) | 0 (0) |
| CoronaVac - 2 doses, n (%) | 58 (4) | 0 (0) | 58 (5) |
| Other vaccines, n (%) | 13 (<1) | 0 (0) | 13 (1) |
