# Supplementary material for: Primary series COVID-19 vaccine effectiveness among health care workers in the country of Georgia, March–December 2021
Source: PLoS One. 2024 Sep 6;19(9):e0307805. doi: 10.1371/journal.pone.0307805 (PMC11379210; doi:10.1371/journal.pone.0307805)
Supplement: Attachment — Submitted filename: Georgia VERespRevcover letter_29Jan2024_POne.docx [file pone.0307805.s003.docx]

29 January 2024

Dear Plos One Editorial Board,

I am pleased to re-submitting our manuscript “Primary Series COVID-19 Vaccine Effectiveness among Health Care Workers in the country of Georgia, March–December 2021” for publication in Plos One. We originally submitted the manuscript last month, but we were mistakenly provided with a review for a different manuscript, and therefore the Plos One staff requested that we resubmit this manuscript as a revision. In fact, though, it has not gone through initial review, and therefore we have no actual response to reviewers to provide.

The manuscript describes results from a prospective cohort study that evaluated the effectiveness of primary COVID-19 vaccine series in preventing symptomatic COVID-19 infection, medically attended infection, and hospitalization in healthcare workers in Georgia.

We found that among a cohort of healthcare workers with high rates of previous infection, primary series vaccine effectiveness (VE) against PCR- or rapid antigen test- confirmed symptomatic SARS-CoV-2 infection was 58% (95%CI: 41; 70) overall; 68% (95%CI: 51; 79) for BNT162b2, and 40% (95%CI: 1; 64) for BBIBP-CorV vaccines . Among previously infected HCWs, VE was 58% (95%CI: 11; 80). VE against medically attended COVID-19 was 52% (95%CI: 28; 68), and VE against hospitalization was 69% (95% CI: 36; 85).

We feel that our manuscript would be particularly relevant for your journal because to date very few studies have evaluated the effectiveness of COVID-19 vaccine in the eastern part of the European region of WHO, an area where uptake of primary vaccine series and booster doses have been quite low. In addition, our methodologically rigorous study, which employed case detection using PCR, rapid antigen tests, and seroconversion, also provides some of the few available VE data for BBIBP-CorV, which is commonly used in the eastern part of the European region. Finally, our study was able to evaluate VE against more severe outcomes, such as medically attended infection and hospitalization. While our results focus on the Delta period, our findings provide local data to underscore the importance of COVID-19 vaccination, even among previously infected individuals, in a region where COVID-19 vaccine uptake continues to lag.

Thank you for considering our manuscript for publication.

Please note that Dr. Khatuna Zakhashvili is not included as a co-author on this manuscript because she has not been actively involved in the study since its inception. She has not been included in other publications and abstract submissions related to this study.

Sincerely,

Mark Katz, MD

Health Emergencies/High Threat Pathogens program

World Health Organization/Regional Office for Europe

Copenhagen, Denmark
