# Supplementary material for: Primary series COVID-19 vaccine effectiveness among health care workers in the country of Georgia, March–December 2021
Source: PLoS One. 2024 Sep 6;19(9):e0307805. doi: 10.1371/journal.pone.0307805 (PMC11379210; doi:10.1371/journal.pone.0307805)
Supplement: Attachment — Submitted filename: POne Reviewer Response Georgia HW Delta_12June2024.docx [file pone.0307805.s004.docx]

12 June 2024

PONE-D-23-33035R1

Primary Series COVID-19 Vaccine Effectiveness among Health Care Workers in the country of Georgia, March–December 2021

PLOS ONE

Dear Dr. Lau,

Thank you and thanks to the reviewer for the helpful comments. We have tried to address every query in our point-by-point response below.

Sincerely,

Mark Katz

High Threat Pathogens Team

Infectious Hazard Management

Health Emergencies Program

WHO Regional Office for Europe

Email: [katzm@who.int](mailto:katzm@who.int)

Reviewer #1: Introduction

Given tha rationale of the study, the introduction section lacks of a brief paragraph describing the precious role of health care workers in tackling COVID-19 pandemic. Moreoever, a further paragraph pointing out the importance of planning, coordinating and carrying out vaccination campaigns, in high-, middle-, and lower-income countries, should be anticipated in this section too.
Doing so, would allow the possibility to briefly state the benefits on the healthcare system (e.g., reduction of the pressure on the health care facilities, reduciton of the infections and overall mortality).
See some examples of references:

[Pascucci D, Nurchis MC, Sapienza M, et al. Evaluation of the Effectiveness and Safety of the BNT162b2 COVID-19 Vaccine in the Vaccination Campaign among the Health Workers of Fondazione Policlinico Universitario Agostino Gemelli IRCCS. Int J Environ Res Public Health. 2021;18(21):11098. Published 2021 Oct 22. doi:10.3390/ijerph182111098]

[Gaio V, Santos AJ, Amaral P, et al. COVID-19 vaccine effectiveness among healthcare workers: a hospital-based cohort study. BMJ Open. 2023;13(5):e068996. Published 2023 May 2. doi:10.1136/bmjopen-2022-068996]

[Arriola CS, Soto G, Westercamp M, Bollinger S, Espinoza A, Grogl M, Llanos-Cuentas A, Matos E, Romero C, Silva M, Smith R, Olson N, Prouty M, Azziz-Baumgartner E, Lessa FC. Effectiveness of Whole-Virus COVID-19 Vaccine among Healthcare Personnel, Lima, Peru. Emerg Infect Dis. 2022 Dec;28(13):S238-S243. doi: 10.3201/eid2813.212477. PMID: 36502444; PMCID: PMC9745240]

Lastly, it is of paramount importance to clearly describe what is the primary aim of the study at the end of the introduction section.

***Response:***

*Thank you for raising these points.*

*We have added language to the first paragraph that describes the critical role of healthcare workers during the COVID-19 pandemic and pandemics in general. In this paragraph we have incorporated 2 of the 3 references suggested by the reviewer. The paragraph reads as follows (Lines 64-86):*

During the COVID pandemic and all pandemics, it is critical to protect the health of HCWs for a number of reasons; ^3–5^ first, in order to ensure the continuous functioning of the healthcare system, a critical essential service in the pandemic response, and one which is particularly challenging in resource-poor healthcare systems in low- and middle-income countries (LMICs).^2^; second, HCWs may have greater likelihood of getting infected with SARS-CoV-2 and other pathogens compared to the general population because of their close contact with infected patients; third, infected HCWs in healthcare settings risk transmitting viruses to vulnerable patients; finally, protecting HCWs is important because of the principle of reciprocity; through their vital front-line role in the pandemic response, HCWs put themselves at risk and also potentially put their households at higher risk for the sake of others.^5^

***Response*:**

*In addition, we have added the following brief paragraph that highlights the importance of planning, coordinating and carrying out vaccination campaigns in high-, middle-, and lower-income countries (Lines 88-90).*

In addition, HCWs play a vital role in the planning, coordination and execution of vaccination campaigns in high-income countries and LMICs. Population-wide COVID-19 vaccination campaigns have been shown to reduce pressure on the healthcare systems.

*Lastly, we have described the primary aim of the study at the end of the introduction section, as follows (line 117-129):*

In order to understand COVID-19 VE in HCWs in Georgia, we conducted a prospective cohort study of COVID-19 VE against symptomatic SARS-CoV-2 infection among HCWs in six hospitals in Georgia.

Materials and methods

It should be useful to better explicit, alongside the inclusion criteria, the exclusion ones.
There is no mention about sample size estimation. How was the sample size computed? Is it a purposive sampling? Please, specify these issues.

***Response:***

*We have added the exclusion criteria, as follows (Lines 145-147):*

We excluded participants who were not eligible for vaccination before their enrolment in the study due to a SARS-CoV-2infection in the previous 120 days, and we excluded participants with unknown vaccination status at the time of enrollment.

*In addition, we have now added the following language to explain sample size calculations, as follows (185-192):*

We conducted sample size estimations prior to enrolment to ensure robust estimates for the primary study objective, COVID -19 VE against symptomatic SARS-CoV-2 infection. We estimated COVID-19 vaccination coverage among HWs in Georgia of 60-90% and a varying incidence of SARS-CoV-2 infection among unvaccinated participants of 0.05-0.2 during a one-year study in order to detect VE between 50-90%. To allow for different scenarios (changes in infection rates in the community, different vaccines), analysis of secondary endpoints, and to account for likely drop-out rate of roughly 15%, 1600 participants were targeted for enrolment in the study. We did not undertake purposive sampling; all eligible HCWs were offered enrolment.

*We also conducted a retrospective calculation in order to confirm that our study had a sufficient statistical power (0.95) for detecting the effect of the primary course vaccination against SARS-CoV-2 infection. In this calculation, which was based on a sample size of 1561 HCWs, vaccine coverage of 57%, a SARS-CoV-2 incidence among the unvaccinated measured at 0.4 SARS-CoV-2 infections per year, and an estimated VE of 58%., we determined that sample size was adequate. We have not added this text to the manuscript, but we have shared it here for the reviewer.*

Discussion

It would be useful to discuss few main implications associated with vaccinating HCWs in light of the results about VE. This could have a significant impact on health systems, thus promoting tailored and targeted health policies should be clearly pointed out.

***Response:***

*Thank you for bringing up this point. We have added the following sentences that address the importance of vaccinating healthcare workers in light of these positive VE findings (Line 456-460):*

Our finding that COVID-19 vaccine prevented nearly two-thirds of symptomatic infections has positive implications for the role of vaccine in protecting the health of HCWs, the health of the patients and improving the resilience of the Georgian healthcare system. These findings underscore the importance of tailored messaging to highlight these points, and health policies to encourage increased COVID-19 vaccine uptake among HCWs.

The Authors are expected to address all the criticisms by the Reviewer. In particular, please state the study objective clearly and add sample size considerations (Reviewer #1).

*We have done our best to address the reviewer’s comments, and in particular we have now clearly stated the study objective and we have added language to explain sample size considerations.*

In additional to the above comments, please address,
1. Serology, please make a clear statement about the ability of and how the serological tests were used to differentiate between SARS-CoV-2 infection, previous COVID-19 vaccination with mRNA and inactivated vaccines.

*We have added the following statement about the use of serology (line 180-182).*

Serology test results were used to identify previous COVID-19 infections, and to identify new infections in a sensitivity analysis that included only unvaccinated HCWs and HCWs who had received mRNA vaccines, as described in the vaccine effectiveness analysis section below.

*In the vaccine effectiveness analysis section we have also expanded the following section to explain our use of serology test results (Lines 219-229):*

In addition, we measured primary series VE against both symptomatic and asymptomatic SARS-CoV-2 infection, measured by a combined endpoint of a PCR-positive test, a RAT-positive test, and/or seroconversion, which we defined as a positive three-month or six-month anti-nucleocapsid antibody test in a participant who was previously seronegative. For participants who seroconverted during the study but did not have symptomatic illness prior to their seroconversion, we estimated the time of asymptomatic infection as halfway between the last negative serological test and the subsequent positive serological test, taking into account a 3-week lag for seroconversion among asymptomatic persons. For participants who had a symptomatic illness prior to their seroconversion, but did not have a positive PCR or RAT test, we assumed that the infection occurred on the date of onset of their symptomatic illness. For this analysis we only evaluated BNT162b2 vaccine effectiveness; we excluded participants who received BBIBP-CorV, as inactivated vaccines can generate the production of anti-nucleocapsid antibodies.

2. Please make an assessment on how the above will affect the main study results especially for inactivated vaccine BBIBP-CorV.

*We have explained, as noted above, that for our secondary analysis of VE that included seroconversion, we excluded participants vaccinated with BBIPV-CorV. For our main analysis of VE against symptomatic VE as documented by PCR and RAT infection, the serology test results were only relevant for defining previous infection. We have further explained the limitation of serology in defining previous infection in the limitations section (Lines 480-491)*

Additionally, while serology was a strength of our study in identifying previous infections and new infections (in those participants who were not vaccinated with inactivated virus vaccines), our anti-nucleocapsid antibody likely did not capture all previous infections, due to a combination of antibody waning, imperfect sensitivity of the assay, and potentially variable production of anti-nucleocapsid antibodies in vaccinated individuals. Furthermore, we were not able to use serology test results to identify previous infections in 86 (5.5%) HCWs who had received their first COVID-19 vaccine more than five days prior to enrolment; however, we believe that by defining previous by a composite of documented PCR and RAT results, combined with serology test results, we captured the nearly all previous infections, and the amount of missed previous infections would be very unlikely to meaningfully impact our results.

3. Results, the abbreviations VE and aVE were used interchangeably. Please harmonize and confirm that adjusted estimates are always presented.

*We have changed the language throughout the document so that only the term VE is used, rather than aVE, and we have explained in the methods section that all VE estimates shared in the results section reflect adjusted VE (Lines 252).*

4. Line 276, there are some typos in the 95% CIs, for example remove “– “ in “(95%CI: 90; –98)” (Line 276), also in lines 277, 283, 292, 328 and others

*Thank you for pointing out these typos. We have gone through the document and corrected all of the typos in the lines pointed out above, and we have made additional corrections to other typos in the document.*
